# Promoting Responsible Use of AI in African Healthcare: Strengthening Patients’ Moral Agency

**DOI:** 10.1007/s41649-025-00357-1

**Published:** 2025-04-25

**Authors:** Edmund Terem Ugar

**Affiliations:** Centre for Africa-China Studies, University of Johannesburg, Johannesburg, South Africa

**Keywords:** Medical machine learning, Social technologies, Healthcare, Afro value systems, Interpersonal relationship, Clinical diagnosis

## Abstract

Machine learning technologies deployed in several sub-Saharan African countries to assist medical practitioners have shown how such technologies can significantly extend the reach of limited medical personnel and equipment resources. However, while I praise the efficiency of these technologies in carrying out medical diagnosis and treatment recommendations, I raise some critical concerns about the normative shift that may occur in their usage in the region. An uncritical use or overreliance on these technologies may threaten shared decision-making between patients and doctors. While shared decision-making is an integral component of patient-centred care in contemporary medicine that must be respected, from a phenomenological perspective, the stakes are higher for sub-Saharan Africans. For Africans, the threat to shared decision-making may negatively impact a significant aspect of the community–interpersonal relationships. I contend that over-relying on these systems for clinical diagnosis and recommendations may diminish the value of interpersonal relationships between patients and doctors. I show how the practice of interpersonal relationships is integral in making a moral agent (to becoming a member of the moral community) within the sub-Saharan African value systems. Finally, this paper seeks to make novel contributions to value-sensitive healthcare/healthcare technology policies, guidelines and regulations within sub-Saharan Africa and countries in the global South that share similar ethical/cultural worldviews.

## Introduction

In the last two decades, there has been a drastic decrease in the mortality rates of patients caused by diseases across different age groups due to the advancements in clinical technologies, which have enabled early detection of diseases and medical diagnosis (Thevenot et al. 2018; Topol 2019). In a study conducted by Balogh et al. (2015), it was shown that clinical diagnostic errors were a significant contributor (10%) to patient mortality and 6–17% of hospital-related complications (c.f. NASEM 2015). Nonetheless, with the growing application of artificial intelligence (AI) and machine learning (ML) techniques in the last three decades, these technologies are now playing a much more significant role in medicine and, increasingly, in public health (Thevenot et al. 2018; Grote and Behrens 2019).

For instance, since its first appearance in the 1980s, Computerised Clinical Decision Systems (CDSS) have become very popular and relevant in healthcare (Osheroff et al. 2007; Salloch and Eriksen 2024). CDSS often provides clinicians and patients with the requisite knowledge and individual-specific information adequately and intelligently filtered to enhance healthcare (Osheroff et al. 2007). CDSS rely extensively on the availability of data to function efficiently. Given the progressiveness of healthcare data storage in the previous and current decades, there has been rapid development of CDSS embedded in machine learning to enhance clinical workflow (London 2018). For example, aspects of healthcare like radiology, ophthalmology and pathology are benefiting from these technologies given their reliance on large quantities of data (see Gulshan et al. 2016; Esteva et al. 2017; Hwang et al. 2019; Bulten et al. 2022). Other surgical and clinical fields in medicine, like dermatology and paediatrics, also profit from this ML-driven CDSS (see Esteva et al. 2017; Liang et al. 2019; Jia et al. 2020).

This development has revamped medical diagnosis, which usually depended on a doctor’s expertise, which in many cases was usually erroneous and sometimes misleading (Oyebode 2006; Dzau 2017; Topol 2019; Miziara and Miziara 2022). However, with the current application of machine learning, especially ML_CDSS, which depends on the patterns from the enormous pull of patient datasets, detecting early disease symptoms has made the healthcare industry efficient (Norgeot et al. 2019; Cioffi et al. 2020). Additionally, machine learning computational technologies, like logistic regression, support vector machine, random forest, naïve Bayes and representational learning, to mention a few, are now applied in the prediction and early detection of diseases. I use two of these examples as use cases in section one. With the accuracy of these algorithms in clinical diagnosis and expertise in predictive abilities, it is *prima facie* that clinicians will benefit immensely from the assistance the technology will provide them in assessing patients’ risks individually on complex datasets.

While these innovations in healthcare are impressive and are a testament to human ingenuity in healthcare technological designs and innovation, there are some normative concerns about these technologies in healthcare, which this paper discusses. One of the normative concerns is that the use of these technologies may create a culture of overreliance on them by medical professionals. I envisage that because of their accuracy and efficiency, medical professionals may begin to depend on them uncritically. If the above happens, their use may limit shared decision-making between patients and medical practitioners and less participation of patients as epistemic and moral agents in their diagnostic and treatment processes. This may lead to healthicisation, medicalisation, or healthcare paternalism (McDougall 2018, 2). Why must these concerns be addressed? I argue that shared-decision making is a prerequisite for efficiency, human-centred medicine, and patient-centred care in modern medicine.

According to the 2017 World Medical Association’s Declaration of Geneva revision of the Hippocratic Oath, it is imperative to respect the autonomy of patients and their contribution to their well-being. Patients’ autonomy in medical decision-making stands at the centre of modern healthcare (Stewart et al. 2006; McDougall 2018, 2). As Whitney (2009) alludes, ‘the general concept of shared decision-making has great appeal. It recognises our conviction that patient and physician are making common cause against illness and suffering and does not relegate the physician to the role of technician…’ (Whitney 2009, 96).

Using the work of Rosalind McDougall (2018) and Sabine Salloch and Andreas Eriksen (2024), I show why it is imperative that patients are empowered to contribute to their diagnosis and treatments, especially in sub-Saharan Africa. On the one hand, McDougall (2018) calls for the view that patients’ individual values must be incorporated into ML-driven clinical decision-making. However, the role of patients has not been specifically clarified beyond expressing their values and preferences. As a result, Salloch and Eriksen (2024) call for the recognition of patients as co-reasoners and epistemic agents when it comes to medical decision-making, on the other hand. I take the arguments presented by these thinkers further in two ways.

First, I claim that we must respect patients as epistemic agents and co-reasoners in the sense that Salloch and Eriksen theorised to empower them to participate in shared decision-making, as alluded to by McDoughall. I further argue that the recognition of patients as epistemic agents/co-reasoners and empowering them to contribute to their diagnosis and treatments has a moral implication, especially in contexts like sub-Saharan Africa. I underscore that this recognition and empowerment of patients to participate in shared decision-making is not just about the needs of the patients. Shared decision-making can act as an important and necessary tool that aids the inclusion of a patient within her moral community in sub-Saharan African cultures. Through shared decision-making, the patient becomes dependent on the medical practitioner both morally and health-wise to exercise their agency of building a friendship of interdependence. As it will become clear in subsequent sections, what makes up a moral agent within this context is one’s ability to strike friendship and interdependence between other members of the community (Metz 2022). On this account, recognising patients as co-reasoners and epistemic agents and empowering them to exercise values like shared decision-making should not be relegated in medical practice due to possible overreliance on machine learning models.

This paper proceeds in the following format. First, an exposition of AI/ML in healthcare is provided. The aim here is to show the influence of AI/ML on healthcare. Second, I show the challenges of using algorithms as co-decision makers in healthcare. Finally, I provide a philosophical argument for the implication of these tools in healthcare, especially within sub-Saharan Africa. I offer a twofold argument for the section above: Firstly, I analyse the general impact of algorithms on shared decision-making in healthcare while attending to some possible objections. Secondly, I narrow the discussion within the sub-Saharan moral community and healthcare system to show the implication of shared decision-making in building the moral agency of the members of this community.

## From Human to Machine Diagnosis and Medical Recommendations

As earlier stated, there has been an increased application of machine learning techniques in healthcare. Clinicians are now encouraging and emphasising the application of machine learning in healthcare decision-making (Grote and Behrens 2019). This is because the full employment of machine learning in healthcare for clinical decision-making can potentially improve how healthcare decisions are carried out. With the application of machine learning in healthcare, decision-making on clinical diagnosis and treatment will be more reliable and quicker (Norgeot et al. 2019; Cioffi et al. 2020). As discussed by Eric Topol (2019), clinical decision-making done by clinicians is currently susceptible to flaws, such as cognitive biases and diagnostic errors. As a result, machine learning techniques have the potential to circumvent these issues and enhance healthcare decision-making capabilities.

Applying machine learning technology in healthcare does not mark the beginning of technological innovation. Expert AIs, designed based on deductive rules given the database, known facts, and how these facts correlate to particular diseases, have been used in clinical diagnosis (Holman and Cookson 1987; Ross and Swelitz 2017). Expert systems that carry out these tasks are usually based on the encoded knowledge of clinical experts. One of the systems that performs this way is the IBM Watson Oncology system. However, IBM’s expert systems are more advanced than previously designed brute expert systems. The IBM Watson Oncology was designed with a combination of automatic text mining of clinical papers and vast logical rules (Ross and Swelitz 2017). However, IBM Watson Oncology had other challenges, such as misdiagnosis (McDougall 2018; Ross and Swelitz 2017).

Contrary to the IBM Watson Oncology, which was designed in a rule-based form with the aim of the algorithm encoding knowledge of experts, machine learning algorithms have been designed to learn how to extract patterns or structures present in labelled or unlabelled data (Norgeot et al. 2019; Esteva et al. 2017; Cioffi et al. 2020). For instance, given its training dataset, these technologies have been designed to read images of X-ray reports to see the patterns embedded in the photo and mark them as normal or abnormal. The learning datasets are labelled according to the disease rating and the diagnostic scale. The algorithm automatically makes an inference that sets its parameters and features, leading to the most accurate predictions based on the original label.

There are two main reasons why machine learning is making waves in healthcare. First, given the increased electronic healthcare data, these data are becoming more accessible to amend and analyse using machine learning techniques (Amrollahi et al. 2022). Additionally, aspects of machine learning algorithms like deep neural networks, which have gained traction, are used to recognise objects in images efficiently (LeCun et al. 2015; Esteva et al. 2017). This has been a long research effort by computer vision software developers for decades (LeCun et al. 2015). With deep neural networks, images can now be accurately labelled.

Deep neural networks are machine learning algorithms which consist of layers and nodes that use simple mathematical operations to carry out specific tasks when the layer is activated, leading to the presence of increasing abstract representations of the input image (LeCun et al. 2015; Esteva et al. 2017). On the one hand, since deep neural networks have many parameters, they need large datasets to achieve good performance. Aspects of healthcare such as radiology, ophthalmology, pathology and dermatology that are image-based have much to benefit from deep neural networks. On the other hand, and most importantly, with the emergence of machine learning, algorithmic decisions can be made more timeously than by humans. As a result, relying on machine learning for decision-making in emergencies will be the utmost and most efficient thing to do. Furthermore, progress has been made in diagnosing sets of diseases from diagnostic images (Esteva et al. 2017; De Fauw et al. 2018). I provide two use cases of machine learning models that have proven effective in healthcare.

### Use Cases of Machine Learning Models in Healthcare

There have been several applications of different machine learning models in healthcare. Models like random forest, representation learning, logistic regression and support vector machines, to mention a few, have been effective in making predictions and treatment recommendations in healthcare. These systems are capable of automating diagnosis and treatment recommendations. Given their roles, in some instances, they are referred to as ML_CDSS. However, I briefly show two use cases in this section—logistic regression and support vector machine.

#### Logistic Regression

Logistic regression is an important aspect of supervised machine learning. It is understood that logistic regression is the baseline of supervised machine learning algorithm classification, which is mainly used with neural networks (Mazzocco and Hussain 2012). As a machine learning model, logistic regression is used to predict categorical-dependent variables and their probability of occurrence (Mazzocco and Hussain 2012). In simple terms, logistic regression determines the likelihood of a particular disease being caused by a specific symptom. This model contains binary variables with data encoded 0, which means negative/no, and 1, which signifies positive/yes (Li et al. 2020).

Logistic regression has been significantly important in automatic diagnostic models in the last decade (Keeley et al. 2017). This model has been applied to diagnose pathologies like dementia (Mazzocco and Hussain 2012). Until now, clinicians have found it challenging to detect dementia using their expert knowledge (Mazzocco and Hussain 2012). This is because of the horrendous complexities of the processes involved in this terrain. However, a logistic regression has been developed to assist in this area. The model generally enhanced performance compared to some other predictive methods that have been employed, such as the Bayesian belief network (Mienye et al. 2020). In other cases, logical regression has been applied to diagnose other cases like sepsis, a life-threatening disease. Sepsis is a disease caused by bacterial infections that enter the blood (Keeley et al. 2017). This disease has resulted in many mortalities among patients. The disease occurs when the substances released into the bloodstream to fight diseases become inflammatory in the body (Keeley et al. 2017; Thompson et al. 2019). Using indicators to analyse latent model feature extraction, logistic regression became effective in predicting the mortality of sepsis.

#### Support Vector Machine

Support vector machine (SVM) is a common machine learning model used in the medical domain for clinical diagnosis. SVM is an aspect of a supervised machine learning classification technique that is classical; yet, it still plays an efficient role in recognising patterns in big data (Suthaharan 2016). SVM does not play the role of regression but a classification role in supervised machine learning. This technique uses a ‘simple mathematical model y = wx + γ and manipulates it to allow linear domain division’ (Suthaharan 2016, 207).

There are two aspects of SVM, which I briefly spell out. The first type of SVM is the linear SVM. Linear SVM applies to data domains that are ‘divided linearly (e.g., straight line or hyperplane) to separate the classes in the original domain’ (Suthaharan 2016, 207). It is worth mentioning that linear SVM, as a method in machine learning, is important in solving problems related to classification and regression. Given its efficiency in making robust predictions from statistical learning theory, it is one of the most widely used machine algorithms in healthcare (Marwala 2014). Contrary to linear SVM, a non-linear SVM, is applied to non-linear data domains or data that cannot be linearly divided (Suthaharan 2016). SVM uses non-linear classification through kernel tricks outside linear classification (Kafai and Eshghi 2017). The kernel trick is important in mapping the non-linear separable input to a high-dimensional space where a hyperplane can separate the determined samples.

As I will show here, SVM has been used to predict many diseases. Given that SVM is a supervised machine learning method, to achieve prediction accuracy and clinical diagnostic efficiency, trained and clean datasets are used to build the model (Gürbüz and Kılıç 2014). SVM has been used to understand and predict breast cancer and diagnose diabetes; the model gained 100% accuracy on both datasets (Mienye et al. 2020). Additionally, SVM has been used to diagnose heart diseases and has shown accuracy and efficiency (Karatsiolis and Schizas 2012; Sun et al. 2017; Misir et al. 2020; Waaijer et al. 2020).

### Why Algorithmic Decision-Making in Healthcare?

As shown in the previous subsection, machine learning models, especially ML_CDSS, show more efficiency and accuracy in making predictions and carrying out diagnoses. In a comparative study, review and meta-analysis of 82 studies, it was evident that physicians and deep learning models in medical image assessments have better performance equivalent to (sometimes better than) physicians (Liu et al. 2019). Furthermore, as earlier spelt out, efficiency and accuracy are critical in medical settings, given issues like high mortality rates due to misdiagnosis and clinical errors. Despite all the procedures that take place when a patient seeks healthcare like information gathering, interviews, physical examination and interpretation of results and second opinions (see NASEM 2015, 32), errors still creep into the results.

Undoubtedly, clinical diagnosis is a collaborative endeavour between medical practitioners (as I will show in the next section, this collaboration is also advised to include patients and other relevant clinicians with expertise). However, despite these processes, clinical errors remain phenomenal in healthcare, as mentioned above. According to the National Academy of Sciences, around 5% of US adults seeking healthcare advice are subjected to diagnostic error. Additionally, diagnostic errors contribute to a 10% mortality rate in patients (NASEM 2015, 11). There are several traceable causes of diagnostic errors. One such cause is uncertainty, which sometimes affects medical diagnoses (Oyebode 2006; Dzau 2017; Topol 2019; Miziara and Miziara 2022). Before a clinician makes a diagnosis, different hypotheses are usually tested to attain certainty. Furthermore, despite the numerous methods of information-gathering activities, some risks are still induced in medical treatment. Other issues, such as time constraints faced by clinicians, constitute the number of diagnostic errors faced (Topol 2019; Miziara and Miziara 2022). For example, some diseases require immediate medical intervention, and clinicians might have limited time to conduct all the necessary tests and assess the available evidence (NASEM 2015, 48).

Given the constraints mentioned above, one can immediately see how machine learning algorithms can contribute to the enhancement of the clinical decision-making capabilities of clinicians. The algorithm can process vast datasets quickly during diagnosis and produce accurate and efficient results. Furthermore, even within a short period, one can infer that the technology might be less susceptible to issues such as cognitive bias. As a result, the algorithm can provide the clinician with additional sources of evidence to enable the clinician to make a well-informed judgement. While these technologies are proving efficient within the healthcare domain in carrying out timely and accurate diagnoses, there are some problems that may stem from their use.

While I find these efficiencies of machine learning models appraisable, there are broadly three concerns which emerge from the use of ML-driven CDSS in medical decision-making, which I engage with in the current research. First, according to some empirical research, it has been discovered that one of the critical concerns of incorporating ML_CDSS is the potential loss of professional autonomy and the challenges of incorporating the clinical workflow of these systems (Lambert et al. 2023). Another concern which pertains to this current research is the loss of patients’ autonomy (McDougall 2018). The third concern is the lack of clarity on the right of patients to refuse diagnoses and treatments that are driven by ML-CDSS (Ploug and Holm 2020). In the subsequent paragraphs, I engage these concerns.

What the above concerns imply is that ML_CDSS may partially replace human clinicians (see Salloch and Eriksen 2024). For example, on the one hand, ML_CDSS integrative systems can gather the data of patients autonomously to present this information to clinicians and document it in their electronic health records. On the contrary, there are also more problematic fully automated ML_CDSS that have the capacity to both supplement the decisions of professions and somewhat change their decision authority (Kempt and Nagel 2022; Salloch and Eriksen 2024). These fully automated ML_CDSS, besides augmenting the decision-making of professionals, to a certain extent, may replace human reasoning in several tasks, like providing more diagnostic services (see Kempt and Nagel 2022; Salloch and Eriksen 2024, 69). Some researchers have suggested that soon, these intelligent systems may ‘replace human ethical decision-making in certain settings’ (Meier et al. 2022, 17). Researchers like Grote and Berens (2019) allude that deploying ML_CDSS may threaten or shift the epistemic authority and norms of medical diagnosis. Their view implies that the normative justification for the decision-making of clinicians might become blurring.

While some researchers are optimistic about the potential of these tools replacing human reasoning and ethical decision-making, others call for a design of ML_CDSS that is supportive of clinicians rather than replacing them. In the next section, using the works of McDougall (2018) and Salloch and Eriksen (2024), I argue that we must not only develop ML_CDSS that are supportive of clinicians but also create a healthcare environment where these tools may be used to empower patients as epistemic and moral agents capable of contributing to their diagnosis and treatments. The reason for such designs, especially in sub-Saharan Africa, is moral significance, as I will show.

## Analysing the Challenges of using Machine Learning Models in Healthcare

To address the problem listed at the end of the previous section, it is important to note that there are two central aspects of medical care for which machine learning systems have been developed or are being developed to cater for—diagnoses and treatments (Sharkey and Sharkey 2013; McDougall 2018; Liu et al. 2018; Esteva et al. 2017). However, machines should not be allowed to make autonomous decisions on the above aspects. As a result, some studies in bioethics call for the autonomous contributions of patients in their medical decision-making on diagnosis and treatments (Emmanuel and Emmanuel 1992; Chin 2002). Patients’ autonomy warrants that they co-participate in the decision-making that concerns their health (Whitney 2009, 96). The works of McDougall (2018) and Salloch and Eriksen (2024) tend to address the restoration of patient’s autonomy in medical decision-making within the context of ML_CDSS application in healthcare. To begin with, Salloch and Eriksen (2024) allude that when it comes to the use of ML_CDSS in healthcare, concepts such as ‘meaningful human control’ or ‘effective human oversight’ (Haselager et al. 2024) must be employed to serve as key ideas to aid the control of the decisive steps in medical decision-making. It is imperative to introduce regulatory frameworks that ensure human agents can overrule and control the machine when necessary (Salloch and Eriksen 2024).

While it is necessary to employ the ‘human in the loop’ it is also clear to specify what human we are referring to. In many instances, when it comes to clinical setups, clinicians are always mentioned as those to take charge of clinical procedures while patients act as consenters (see Hille et al. 2023). However, Salloch and Eriksen (2024) argue that patients must be introduced into the loop as active participants, not merely as consenters but as co-reasoners. Building on Onora O’Neill’s (1989, 82) notion of ‘fellow workers’, Salloch and Eriksen (2024) call for patients to become fellow workers in medical spaces.

In a brief sense, just to shed light to the reader, O’Neill’s (2015, 13) notion of fellow workers is informed by Immanuel Kant’s argument of the concept of ‘reason’—that which ‘presupposes plurality-without-preestablished-harmony’. What this implies is that reason must be open to accepting valid claims and contestation and should not be based on a one-sided power where force is used to back-up claims rather than reason. O’Neill writes: ‘Only those who try to think from the standpoint of everyone else and strive to listen to and interpret others and to see the point of their contributions are genuinely aiming to be ‘’fellow workers’’ and to avoid maxims to which others cannot agree’ (O'Neill 1989, 26).

What the above exposition in the contexts of Salloch and Eriksen (2024) means is that, as fellow workers or co-reasoners, patients should be ‘empowered’ to have the requisite skills to communicate their preferences during treatments (see Emmanuel and Emmanuel 1992, 2222; Elwyn et al. 2010, 971; Salloch and Eriksen 2024). Empowering patients in this context means providing them with the environment to become epistemic responsible agents who can communicate their valid cognitive claims. In this sense, ML_CDSS should be used as discussion prompts to instigate or prompt doctors and patients to engage as co-reasoners and fellow workers in disseminating and articulating the values of patients and treatment goals (see Zhou and Danks 2020, 158). This is because the role of fellow workers is epistemic with moral implications, which fosters an interaction where one is listened to and interpreted in such a way that their contributions are made clear (O’Neill 1989, 26).

Building on the above, one can see the connection raised in the concerns of McDougall (2018) that ML_CDSS may obscure doctor-patient relationships in terms of shared decision-making, especially when the technologies are no longer used as discussion prompts but overly relied on to rank diagnosis and treatments options. When there is shared decision-making, ‘the physician can have a responsibility to explore, together with the patient, the values by which alternatives should be evaluated’ (Frankel et al. 2009, 39). Nonetheless, with the advancement of AI or machine learning technology in healthcare, McDougall worries that the above integral aspect of care could be threatened. As I will show shortly, if shared decision-making is threatened, there are some moral implications that may arise for sub-Saharan Africans.

As Rosalind McDougall (2018, 2-3) writes:

AI systems that recommend treatment options present a potential threat to shared decision-making because the individual patient’s values do not drive the ranking of treatment options…so there are two main reasons to see these types of AI systems as a potential threat to shared decision-making. First, the values driving the treatment rankings are not specific to the individual patient…second, these types of AI systems currently do not encourage doctors and patients to recognise treatment decision-making as value-laden. There is a danger that the computer is seen as figuring out the correct answer.

McDougall (2018) shows why AI systems may become a potential threat to shared decision-making. She argues that individual values are not usually considered during the treatment ranking process. Within the healthcare domain, diverse value systems should be respected. This is on the grounds that patients and their caregivers come with different values, given their different worldviews, cultures and the communities they come from. Current designs of AI are yet to learn how to encourage clinicians to recognise the values embedded in treatment decision-making (Friedman et al. 2008; van de Poel 2020; Ugar and Malele 2024).

At this point, it is becoming clearer that there is an interlink between the concerns of McDougall (2018) and Salloch and Eriksen (2024). The reason why shared decision-making must not be obscured in medical settings, in McDougall’s terms, is because patients are not just objects of medical treatment. Patients are fellow workers or co-reasoners with the epistemic capacities to contribute to their diagnosis and treatments in the way theorised by Salloch and Eriksen (2024). As a result, the values (which are diverse) of patients must be respected. One way to respect these values is through shared decision-making.

Theorists like Dignum (2017, 4702) allude that ‘each individual and socio-cultural environment prioritises different moral and societal values’; however, there are ‘prevailing values or community values’. For instance, sub-Saharan Africans, as I will show shortly, would value shared decision-making in healthcare not for its own sake but due to its instrumental role in building the moral agency of patients. The value of shared decision-making is important because of the moral role it plays—building interdependence and interpersonal relationships between the caregiver and the patients. Values such as interpersonal relationships and interdependence are imperative for those within the sub-Saharan African moral space to develop their moral agency and become full persons. These values of interdependence and interpersonal relationships can be significantly attained through shared decision-making. However, at this point, some possible objections may be raised against my concerns here.

First, a critic may argue that machine learning models in healthcare do not make decisions. Medical decisions are made by clinicians, and the machine learning systems only make recommendations to the practitioners. If this is the case, it follows that these systems cannot threaten shared decision-making in healthcare. Furthermore, the critic may argue that if machine learning models are effective and accurate in carrying out diagnoses and making the best treatment recommendations, why should these effectiveness and accuracy be problematised?

The above possible objection is centred on the oblivion of the current human–machine relationships. To respond to the above possible objection, I turn to Don Ihde’s (1990) and Peter-Paul Verbeek’s (2005; 2008) post-phenomenology discourses. Don Ihde (1990) argues that the human-world relationship is mediated through technology. In Ihde’s theorisation, human perceptions and actions are mediated by technologies. These tools provide us with avenues to construct and reconstruct the world.

In postphenomenological scholarship, technology is theorised as a tool that aids us in shaping our human perception and interpretation of reality (Verbeek 2005; 2008). These tools assist us in making moral decisions. In the current technological culture, humans and technology are intertwined in such a way that they are not in a separate existence anymore but assistive in shaping each other (Verbeek 2008). As Aaron Smith (2003) claims, humans no longer visibly occupy the prime mover status anymore when it comes to human-technology relations—this status is now concealed even though it is still there. It becomes an unnecessary belabour to hold on to the prime mover status of humans in the above relations as it may distort the moral importance of technology. The point here is that humans should not be oblivious or dismissive of the role technology plays in shaping their lifeworlds. Technologies are constantly shaping and reshaping our moral subjectivity. Given that ML_CDSS are becoming better than human clinicians in terms of diagnosis and treatment recommendations, the possibility that these technologies will begin to make autonomous decisions in healthcare regarding diagnosis and treatment cannot be dismissed at face value. This is because these technologies mediate our experiences with the world (doctor-patient) within this context—they help doctors make decisions with moral implications, as spelt out in the first section on page 6. As spelt out by Kempt and Nagel (2022) and Meier et al. (2022), autonomous ML_CDSS alters professional decision-making; in some instances, it provides further diagnosis. We can immediately see how these technologies are part of our lifeworlds and participate in our moral choices. As a result, it is necessary and imperative that the concerns raised here are taken seriously to mitigate possible issues that may arise from their usage rather than being dismissive.

Second, another critic might point out that my claim that shared decision-making may affect the moral status or agency of sub-Saharan Africans (as it may limit their opportunity to build interpersonal relationships and interdependence) encloses members of this geography within the *myth of unanimity.* The critic may further claim that sub-Saharan Africans are diverse people and should not be homogenised as though they were a single unit with the same practices.

My view that sub-Saharan Africans are communal people with shared values of interdependence and building interpersonal relationships should not be understood as described by the critic above. I am aware that members of this political geography of sub-Saharan Africa are diverse in terms of worldviews, practices and cultures. However, despite their diversity, interpersonal relationships and interdependence are the dominant values that cut across this geography (Metz 2015). As I will show in the next section, building the values of interdependence and interpersonal relationships characterises one as a moral agent or a person within this clime. When shared decision-making in healthcare is threatened, this integral aspect of sub-Saharan Africans is also threatened.

## The Implications of Machine Learning ‘Decision-Making’ on Human Agency

In this section, I show that the possible low inclusion of patients in shared decision-making in healthcare due to the rise of machine learning algorithms may obscure an integral aspect of sub-Saharan African values—the value of interpersonal relationships between medical practitioners and patients. I argue that patients seeking healthcare services are persons, and how they contribute to their treatment through their interaction with their practitioners plays a significant role in making their moral agencies as persons in a community. To make my argument succinct and clear, I begin by showing what it means to be a moral agent/person in the sub-Sahel. Second, I show how machine learning *decision-makers* may impede patients from attaining their full moral agency.

In African philosophical discourse, the question of what constitutes personhood is engaged with given the relevance of personhood in shaping the moral landscape of those in the region. Personhood goes beyond African philosophical discourse to discourses in environmental ethics, bioethics (like in the current discourse) and animal ethics. In these discourses, the notion of personhood is engaged here to differentiate an entity that is a human being from one that is a person (Behrens 2011, 104). The African philosopher Dismas Masolo (2010, 154) contends that ‘being a person and being a human being is not the same thing’. While a human being belongs to the homo sapiens species, a person is one who is ‘born human but becomes a person’ (Masolo 2010, 154).

The notion of personhood signifies four things. First, a person is a being with moral status—a being owed moral treatment for their own sake (Metz 2017). Second, a person also denotes a being that is a moral agent—one who also owes others moral treatment for their own sake (Behrens 2011, 108). The third understanding of personhood signifies a being with self-awareness and rationality (Warren 1973). Last, the term person also means a being who is virtuous (Masolo 2010). In sub-Saharan Africa, the notion of personhood captures all four; however, the focus is placed more on the conceptions that are normative rather than descriptive.

As it will become clearer shortly, sub-Saharan African thinkers conceive the metaphysical characteristics of human beings as necessary but not sufficient in defining a person. Sub-Saharan Africans consider personhood to go beyond one’s psychological traits—traits like rationality, autonomy, free will and others (Ikuenobe 2016; Manzini 2018). In sub-Saharan Africa, a person is not a lone entity in the Cartesian ‘Cogito’ or ‘thinking I’ but a being within a community where the being exists. As it becomes more apparent, personhood is defined based on normative criteria, which include communal recognition, interdependence and interpersonal relationships rather than psychological, metaphysical or physical traits (Wiredu 1996).

For example, in Kwame Gyekye’s (1992, 111) words,

‘When an individual appears in his conduct to be wicked, bad, ungenerous, cruel, selfish, the Akan would say of that individual that “he is not a human person”. On the contrary, an individual can be referred to as a person when ‘the judgment, “he is a person”, is used normatively to signify that “he has good character”, “he is respectful-not troublesome”, “he is kind”, “he has respect for others”, “he is humble”’

In sub-Saharan Africa, members of the community interrelate as one body such that every individual is viewed from the lens of the collective. Considering the assertion of McDougall (2018) that machine learning decision-making systems may thwart shared decision-making, especially the patients’ autonomy to make informed decisions, how that may impact individual interactions becomes clear. Although, in McDougall’s contention, the challenge is that the system infringes on the patient’s autonomy, the African understanding of this problem is more profound. Within the sub-Saharan African moral lens, it is not the patient’s autonomy per se that is infringed upon that is of paramount concern, but the role autonomy plays—the freedom to relate with the other. What is crucial is that ML_CDSS may infringe on the patient’s ability to relate with the doctor as she should in a communitarian setting. Given this challenge, it becomes clear why patients must be allowed to build relationships with their caregivers to maximise their moral agency, communicate their preferences (Emmanuel and Emmanuel 1992) and express their personhood. Furthermore, as underscored by Salloch and Eriksen (2024), it is imperative that patients are respected as epistemic agents and co-reasoners. Thus, patients must be empowered to engage and interact with their practitioners when they can. But why is this important for sub-Saharan Africans?

To be a person in sub-Saharan societies, possessing characteristics like autonomy, consciousness and rationality are secondary. Rather than being descriptive, personhood is defined from a normative perspective. For instance, Afro-communitarian thinkers like Ifeanyi Menkiti (1984, 181) clarify the above point further. Menkiti argues that in sub-Saharan Africa, the understanding of personhood prioritises ‘the duties which individuals owe to the collective…’. As a result, an individual ‘could satisfy all the properties requisite for metaphysical personhood and lack all the properties requisite for moral personhood’ (Beauchamp 1999, 310). The point here is that there is a distinction between metaphysical and moral personhood. The former depends on descriptive criteria; the latter is more normative and depends on normative criteria. The latter understanding is more prominent in sub-Saharan Africa.

This understanding of personhood, the normative account, is espoused by Afro-relational moral theorist Thaddeus Metz. According to Metz (2022), Africans relate with each other by identifying with each other and being in solidarity with one another. The concepts ‘identity and solidarity’ emerge from the harmonious ways sub-Saharans live together. Here, harmony is explained by theorists like Mbiti in aphorisms like ‘I am because you are, since you are, I am’, or ‘a person is a person through other people’ (Mbiti 1970, 141). When sub-Saharan Africans live in harmony, they become sympathetic towards each other (Mokgoro 1998, 17). The South African theologian Desmond Tutu (1999, 35) alludes that being in a harmonious relationship in sub-Saharan Africa implies being a party to the affairs of the group. By being a party to the affairs of the group, one participates in communal activities that define their personhood to be elevated from a human being to a person. Tutu conceives personhood as sharing a way of life and belonging to a community. It must be stated clearly that the African theorists that I have mentioned here do not all completely agree with the criteria that make up a moral person. However, while they have divergent views, the claim that the most important criteria for personhood in Africa (interdependence and building interpersonal relationships) is consistent in all their works. Given that my focus is on these criteria, it is plausible to focus on this area of convergence in their philosophical analysis of personhood.

According to Metz (2022), the notion of identity and solidarity comes from the notion of living harmoniously in sub-Saharan Africa. Metz claims that conditions for interpersonal relationships in sub-Saharan Africa, like identity and solidarity, are prerequisites. Identity implies being ‘part of the whole, being close, participating, sharing a way of life, belonging, and thinking of oneself as bound to others’ (Metz 2022, 147). On the contrary, exhibiting solidarity implies ‘achieving the good of all, being sympathetic, sharing, promoting the common good, engaging in service and being committed to others’ good…caring for others’ quality of life’ (Metz 2022, 147).

To live in solidarity with one another means being aware of what goes on with others—understanding how they feel, expressing themselves, understanding their unsaid concerns and worries and knowing the tiny details about them. Being in solidarity implies being empathetic towards the pain of others, being intuitive to their feelings, sympathising with others and celebrating with them. To be in solidarity with others means to journey with them to improve their biological, psychological and social life. This is precisely what shared decision-making in healthcare ought to aim at—cementing the notion of identity and solidarity (as spelt here) within the healthcare set-up.

Identity and solidarity grounds moral status in sub-Saharan Africa (Metz 2022). An individual who can exercise the conditions of identity and solidarity is considered a subject of communal relationships and can be a party to friendly living. A subject of communal relationships is one with the capacity to relate with other community members. It ‘involves identifying with others and exhibiting solidarity with them…think of oneself as “we”, corporate with others, help others, and act for their sake out of sympathy’ (Oyowe 2013, 105). Contrary to the above, a being can also be an object of a communal relationship. Objects of communal relationships consider themselves as ‘we’, and they sympathetically let others act for them for their own sake. Beings can be both objects and subjects of communal relationships. Beings that are both objects and subjects of communal relationships are beings with full moral status, like human beings. Beings with partial moral status are beings that are objects of communal relationships.

The above exposition shows the centrality of the notion of personhood in African moral discourse, given the fundamental role it plays in African philosophical thoughts. As it has become clear, personhood is based on one’s moral responsibility. When the status is conferred on an individual, they must live by the social expectations the community has of them. This responsibility includes being a party to communal relationships as a subject and object of that relationship, as exposed above. This process is not static but continuous. Thus, being a person is not to ‘describe a human being with body and mind but also an individual who indicates by his action that he can accept and meet certain standards of social responsibility to achieve recognition’ (Ikuenobe 2016, 58). Given the above, the individual is conferred the status of personhood of which she has to sustain through constant interactions with the rest of the community members, including those within healthcare spaces. Moral treatment is not only owed to her, but she also assumes the responsibility of a moral agent by ensuring that she treats others right.

### Reconsidering the Role of Machine Learning Models in Shared Decision-Making

In the previous section, I showed what personhood or human agency means for sub-Saharan Africans. I argued that being a moral agent in sub-Saharan Africa implies being able to enter into a communal relationship of identity and solidarity with members of the community. As an agent, you owe others the responsibility to strike friendship with them as a subject of that relationship, and you are to avail yourself to be related to as an object of this relationship. It is this relationship that equips an individual to be virtuous. Against this backdrop, I argue in this section that a possible overreliance on machine learning techniques for decision-making in healthcare stands a chance to thwart this vital aspect of Africans within the healthcare sector.

As stated earlier, shared decision-making is an essential aspect of healthcare treatment. This is because shared decision-making is imperative for inclusive treatment remedies. This approach recognises the view that patients and practitioners are making common causes to the healing of the patient rather than the physician taking the role of a technician. Additionally, as Salloch and Eriksen (2024) spelt out, patients are not just objects but beings with agency and reasoning capabilities; they must be treated as agents (both epistemic and moral). As a result, clinicians must be mindful that the possible over-dependence or reliance on machine learning to make patient treatment recommendations may relegate the patient to becoming an observer in their treatment process. This is because the shared decision-making between the patient and the physician might be lost, given that the technology ranks treatment options in order of priority. Given this ranking, the physician might be obliged to follow the treatment options with the most plausible evidence to remedy the illness without constantly consulting the patients to know their preferences and possible contributions. As stated earlier in my response to the possible objection, according to postphenomenologists like Ihde (1990) and Verbeek (2005; 2008), technologies tend to shape some of our decisions—which I agree. Technologies mediate some of our moral decisions. Furthermore, the economic utility of applying technology to healthcare may also shape how decisions are carried out within this domain in the future. As a result, this paper raises justified concerns.

Suppose we get into a future where the above becomes a reality. In that case, patients will be forced to embrace the treatment remedies the machine learning models provide against their wishes—even though these recommendations may be ‘accurate’ and ‘efficient’. This is because the machines operate from an evidence-based perspective. However, it is pertinent to understand that patients have values, and their treatment ought to be shaped and tailored in line with those values rather than what is considered ‘efficient’ or ‘accurate’. For example, a technology that advances longevity is efficient and accurate, but it does not mean patients who receive treatments from such systems value longevity. As a result, there should always be constant information sharing between the patients and their physicians about their preferences, values and worldviews. In the instances where technologies are involved, they should be used in sensitivity to the values of the patients. The physician ought to explore with the patient to identify the best treatment remedies that mirror the patient’s values regardless of the tools’ recommendations. While this is crucial as it maintains the patient's autonomy in the treatment process, the stakes are high for sub-Saharan Africans.

For sub-Saharan Africans, the process of information exchanges between the doctor and the patient creates an environment of interpersonal relationships. Here, the doctor and patient form a friendship bond, which makes them both virtuous and builds their moral agency. Within the healthcare sector, given that, in some instances, patients are isolated from the rest of the world, the physician offers the opportunity for the patients to have a relationship with them, be cared for, be engaged with, and be respected as an epistemic and moral agent. Additionally, this goes both ways—in relating with the patient, the physician also builds their moral agency. The point is that the patient’s relationship with the physician is crucial in sub-Saharan Africa. This relationship includes opportunities for patients to contribute to their treatment and share their values, worldviews and preferences with the physician. In the instance where machines obstruct this process—the process of shared decision-making, it changes or obscures possibilities for interpersonal relationships between doctors and patients. Such obstruction has normative consequences in locales like sub-Saharan Africa. This is because it does not advance opportunities for patients to exercise their full moral agency or become moral agents. Given this challenge, physicians must understand contextual relevance while depending on or relying on machine learning tools to rank treatment options and remedies. Physicians must understand the importance of always including patients in their treatment process, especially in places like sub-Saharan Africa, where such a gesture has moral implications in building the virtue of patients as moral agency.

## Conclusion

This paper argued that the possible overreliance on machine learning models to rank treatment options in healthcare due to their diagnostic efficiencies and accuracies poses a threat to the shared decision-making of patients and doctors in the patient’s processes. While the potential obscurity of shared decision-making thwarts the autonomy of patients in contributing to their treatment by detailing their values, preferences, and worldviews to the practitioner, in climes like sub-Saharan Africa, it goes beyond impeding individual autonomy. Obstructing shared decision-making in the sub-Sahel would not allow patients to exercise their moral agency or virtue. This is because patients may not be opportune to create interpersonal relationships with physicians. Creating interpersonal relationships is imperative for patients to be virtuous within this context. Given the inability to relate with others, especially in severe health conditions and isolative states, it is necessary that patients can relate with their physician, as the physician may be the only person they can interact with. This paper makes a novel contribution to the literature on medical ethics and value-sensitive designs of healthcare social technologies. The paper makes the above contribution by pointing out some of the ethical and cultural problems and challenges that may arise from a possible overreliance on machine learning decision-making systems to carry out diagnoses and make recommendations for treatment remedies in order of hierarchy and necessity. While technological innovations in healthcare are plausible and necessary, it is imperative that we consider the importance of value-sensitive use of these technologies.

## References

[CR1] Amrollahi, F., S.P. Shashikumar, A.L. Holder, and S. Nemati. 2022. Leveraging clinical data across healthcare institutions for continual learning of predictive risk models. *Scientific Reports* 12 (1): 8380. 10.1038/s41598-022-12497-7. 35590018 10.1038/s41598-022-12497-7 PMC9117839

[CR2] Balogh, E., B. Miller, and J. Ball. 2015. *Improving diagnosis in healthcare*. Washington DC: The National Academies Press. 26803862

[CR3] Beauchamp, T. 1999. The failure of theories of personhood. *Kennedy Institute of Ethics Journal* 9 (4): 309–324. 10.1353/ken.1999.0023. 11657914 10.1353/ken.1999.0023

[CR4] Behrens, Kevin G. 2011. Two ‘normative’ conceptions of personhood. *Quest* 25 (1–2): 103–118. https://www.quest-journal.net/2011.htm. Accessed 21 Apr 2025.

[CR5] Bulten, W., K. Kartasalo, P.-H.C. Chen, P. Ström, H. Pinckaers, K. Nagpal, Y. Cai, D.F. Steiner, H. van Boven, R. Vink, et al. 2022. Artificial intelligence for diagnosis and Gleason grading of prostate cancer: The PANDA challenge. *Nature Medicine* 28 (1): 154–163. 10.1038/s41591-021-01620-2. 10.1038/s41591-021-01620-2 PMC8799467 35027755

[CR6] Chin, J.J. 2002. Doctor-patient relationship: From medical paternalism to enhanced autonomy. *Singapore Medical Journal* 43 (3): 152–155. https://pubmed.ncbi.nlm.nih.gov/12005343/. Accessed 21 Apr 2025. 12005343

[CR7] Cioffi, R., M. Travaglioni, G. Piscitelli, A. Petrillo, and F. De Felice. 2020. Artificial intelligence and machine learning applications in smart production: Progress, trends, and directions. *Sustainability* 12 (2): 492. 10.3390/su12020492.

[CR8] De Fauw, J., R. Ledsam, B. Romera-Paredes, et al. 2018. Clinically applicable deep learning for diagnosis and referral in retinal disease. *Nature Medicine* 24 (9): 1342–1350. 10.1038/s41591-018-0107-6. 10.1038/s41591-018-0107-6 30104768

[CR9] Dignum, Virginia. 2017. Responsible Autonomy. In *Proceedings of the Twenty-Sixth International Joint Conference on Artificial Intelligence AI and autonomy track*, 4698–4704. 10.24963/ijcai.2017/655.

[CR10] Dzau, V. 2017. *Diagnostic error: Under-recognised problem*. Washington DC: National Academy of Medicine.

[CR11] Elwyn, G., D. Frosch, R. Thomson, et al. 2010. Shared decision-making: A model for clinical practice. *Journal of General Internal Medicine* 27 (10): 1361–1367. 10.1007/s11606-012-2077-6. 10.1007/s11606-012-2077-6 PMC3445676 22618581

[CR12] Emanuel, J., and L. Emanuel. 1992. Four models of the physician-patient relationship. *JAMA* 267 (16): 2221–2226. 10.1001/jama.1992.03480160079038. 1556799

[CR13] Esteva, A., B. Kuprel, R. Novoa, J. Ko, S. Swetter, H. Blau, and S. Thrun. 2017. Dermatologist-level classification of skin cancer with deep neural networks. *Nature* 342 (7639): 115–118. 10.1038/nature21056. 10.1038/nature21056 PMC8382232 28117445

[CR14] Frankel, R., A. Goldworth, V. Rorty, A. Silverman, et al. (eds.). 2009. *Ethical dilemmas in paediatrics: Cases and commentaries*. New York: Cambridge University Press.

[CR15] Friedman, B., P. Kahn, and A. Borning. 2008. Value-sensitive design and information systems. In *The handbook of information and computer ethics*, eds. E. Himma and T. Tavani, 69–101. Hoboken, NJ: John Wiley & Sons.

[CR16] Grote, T., and P. Berens. 2019. On the ethics of algorithm decision-making in healthcare. *Journal of Medical Ethics* 46 (3): 205–211. 10.1136/medethics-2019-105586. 31748206 10.1136/medethics-2019-105586 PMC7042960

[CR17] Gulshan, V., L. Peng, M. Coram, M. Stumpe, D. Wu, A. Narayanaswamy, S. Venugopalan, K. Widner, T. Madams, J. Cuadros, R. Kim, R. Raman, P. Nelson, J. Mega, and D. Webster. 2016. Development and validation of a deep learning algorithm for detection of diabetic retinopathy in retina fundus photographs. *JAMA* 316 (22): 2402–2410. 10.1001/jama.2016.17216. 27898976 10.1001/jama.2016.17216

[CR18] Gürbüz, E., and E. Kılıç. 2014. A new adaptive support vector machine for diagnosis of diseases. *Expert Systems* 31 (5): 389–397. 10.1111/exsy.12051.

[CR19] Gyekye, Kwame. 1992. Person and Community in African Thought. In *Person and Community, Ghanaian Philosophical Studies, I*, eds. Kwasi Wiredu and Kwame Gyekye, 101–122. Washington, DC: Council for Research in Values and Philosophy.

[CR20] Haselager, P., H. Schraffenberger, S. Thill, S. Fischer, P. Lanillos, S. van de Groes, and M. van Hooff. 2024. Reflection machines: Supporting effective human oversight over medical decision support systems. *Cambridge Quarterly of Healthcare Ethics* 33 (3): 380–389. 10.1017/S0963180122000718. 36624620 10.1017/S0963180122000718

[CR21] Hille, E. M., P. Hummel, and M. Braun. 2023. Meaningful human control over AI for health? A review. *Journal of Medical Ethics*, 20 September 2023. 10.1136/jme-2023-109095. 10.1136/jme-2023-109095 PMC13151516 37730418

[CR22] Holman, J.G., and M.J. Cookson. 1987. Expert systems for medical applications. *Journal of Medical Engineering & Technology* 11 (4): 151–159. 10.3109/03091908709008986. 3316658 10.3109/03091908709008986

[CR23] Hwang, E.J., J.G. Nam, W.H. Lim, S.J. Park, Y.S. Jeong, J.H. Kang, E.K. Hong, T.M. Kim, J.M. Goo, S. Park, et al. 2019. Deep learning for chest radiograph diagnosis in the emergency department. *Radiology* 293 (3): 573–580. 10.1148/radiol.2019191225. 31638490 10.1148/radiol.2019191225

[CR24] Ihde, D. 1990. *Technology and the lifeworld*. Bloomington: Indiana University Press.

[CR25] Ikuenobe, P. 2016. The communal basis for moral dignity: An African perspective. *Philosophical Papers* 45 (3): 437–469. 10.1080/05568641.2016.1245833.

[CR26] Jia, P., P. Jia, J. Chen, P. Zhao, and M. Zhang. 2020. The effects of clinical decision support systems on insulin use: A systematic review. *Journal of Evaluation in Clinical Practice* 26 (4): 1292–1301. 10.1111/jep.13291. 31782586 10.1111/jep.13291

[CR27] Kafai, M., and K. Eshghi. 2017. CROification: Accurate kernel classification with the efficiency of sparse linear SVM. *IEEE Transactions on Pattern Analysis and Machine Intelligence* 41 (1): 34–48. 10.1109/tpami.2017.2785313. 29990038 10.1109/TPAMI.2017.2785313

[CR28] Karatsiolis, S., and C.N. Schizas. 2012. Region based Support Vector Machine algorithm for medical diagnosis on Pima Indian Diabetes dataset. In *2012 IEEE 12th International Conference on Bioinformatics & Bioengineering (BIBE)*, 139–144. IEEE.

[CR29] Keeley, A., P. Hine, and E. Nsutebu. 2017. The recognition and management of sepsis and septic shock: A guide for non-intensivists. *Postgraduate Medical Journal* 93 (1104): 626–634. 10.1136/postgradmedj-2016-134519. 28756405 10.1136/postgradmedj-2016-134519

[CR30] Kempt, H., and S.K. Nagel. 2022. Responsibility, second opinions and peer-disagreement: Ethical and epistemological challenges of using AI in clinical diagnostic contexts. *Journal of Medical Ethics* 48 (4): 222–229. 10.1136/medethics-2021-107440. 34907006 10.1136/medethics-2021-107440

[CR31] Lambert, S.I., M. Madi, S. Sopka, A. Lenes, H. Stange, C. Buszello, and A. Stephan. 2023. An integrative review on the acceptance of artificial intelligence among healthcare professionals in hospitals. *NPJ Digital Medicine* 6 (1): 111. 10.1038/s41746-023-00852-5. 37301946 10.1038/s41746-023-00852-5 PMC10257646

[CR32] LeCun, Y., Y. Bengio, and G. Hinton. 2015. Deep learning. *Nature* 521: 436–444. 10.1038/nature14539. 26017442 10.1038/nature14539

[CR33] Li, J.P., A.U. Haq, S.U. Din, J. Khan, A. Khan, and A. Saboor. 2020. Heart disease identification method using machine learning classification in e-healthcare. *IEEE Access* 8: 107562–107582. 10.1109/ACCESS.2020.3001149.

[CR34] Liang, H., B.Y. Tsui, H. Ni, C.C.S. Valentim, S.L. Baxter, G. Liu, W. Cai, D.S. Kermany, X. Sun, J. Chen, et al. 2019. Evaluation and accurate diagnoses of pediatric diseases using artificial intelligence. *Nature Medicine* 25 (3): 433–438. 10.1038/s41591-018-0335-9. 10.1038/s41591-018-0335-9 30742121

[CR35] Liu, X., A. Keane, and K. Denniston. 2018. Time to regenerate: The doctor in the age of artificial intelligence. *Journal of the Royal Society of Medicine* 111 (4): 113–116. 10.1177/0141076818762648. 29648509 10.1177/0141076818762648 PMC5900836

[CR36] Liu, X., L. Faes, A.U. Kale, S.K. Wagner, D.J. Fu, A. Bruynseels, T. Mahendiran, G. Moraes, M. Shamdas, C. Kern, et al. 2019. A comparison of deep learning performance against health-care professionals in detecting diseases from medical imaging: A systematic review and meta-analysis. *The Lancet. Digital Health* 1 (6): e271–e297. 10.1016/S2589-7500(19)30123-2. 33323251 10.1016/S2589-7500(19)30123-2

[CR37] London, A. J. 2018. Groundhog day for medical artificial intelligence. *The Hastings Center Report* 48 (3):inside back cover. 10.1002/hast.842. 10.1002/hast.842 29806902

[CR38] Manzini, Z. 2018. Menkiti normative conception of personhood as gendered, ableist, and anti-queer. *South African Journal of Philosophy* 37 (1): 18–33. 10.1080/02580136.2017.1405510.

[CR39] Marwala, T. 2014. *Artificial intelligence techniques for rational decision making*. Springer.

[CR40] Masolo, D. 2010. *Self and community in a changing world*. Bloomington: Indiana University Press.

[CR41] Maziara, I., and C. Maziara. 2022. Medical errors, medical negligence and defensive medicine: A narrative review. *Clinics* 77 (100053): 1–5. 10.1016/j.clinsp.2022.100053. 10.1016/j.clinsp.2022.100053 PMC9160317 35640458

[CR42] Mazzocco, T., and A. Hussain. 2012. Novel logistic regression models to aid the diagnosis of dementia. *Expert Systems with Applications* 39 (3): 3356–3361. 10.1016/j.eswa.2011.09.023.

[CR43] Mbiti, J. 1970. *African religions and philosophy*. London: Heinemann.

[CR44] McDougall, R. 2018. Computer knows best? The need for value-flexibility in medical AI. *Journal of Medical Ethics* 45 (3): 156–160. 10.1136/medethics-2018-105118. 30467198 10.1136/medethics-2018-105118

[CR45] Meier, L.J., A. Hein, K. Diepold, and A. Buyx. 2022. Algorithms for ethical decision-making in the clinic: A proof of concept. *American Journal of Bioethics* 22 (7): 4–20. 10.1080/15265161.2022.2040647. 10.1080/15265161.2022.2040647 35293841

[CR46] Menkiti, I. 1984. Person and community in African traditional thought. In *African philosophy: An introduction*, ed. R. Wright, 171–181. Lanham: University Press of America.

[CR47] Metz, T. 2015. How the West was one: The Western as individualist, the African as communitarian. *Education, Philosophy, and Theory* 47 (11): 1175–1184. 10.1080/00131857.2014.991502.

[CR48] Metz, T. 2017. How to ground animal rights on African values: A reply to Horsthemke. *Journal of Animal Ethics* 7 (2): 163–174. 10.5406/janimalethics.7.2.0163.

[CR49] Metz, T. 2022. *A relational moral theory: African ethics in and beyond the continent*. London: Oxford University Press.

[CR50] Mienye, I.D., Y. Sun, and Z. Wang. 2020. Improved sparse autoencoder based artificial neural network approach for prediction of heart disease. *Informatics in Medicine Unlocked* 18: 100307. 10.1016/j.imu.2020.100307.

[CR51] Misir, S., C. Hepokur, Y. Aliyazicioglu, and F.J. Enguita. 2020. Circular RNAs serve as miRNA sponges in breast cancer. *Breast Cancer* 27 (6): 1048–1057. 10.1007/s12282-020-01140-w. 32715419 10.1007/s12282-020-01140-w

[CR52] Mokgoro, Y. 1998. Ubuntu and the law in South Africa. *Potchefstroom Electronic Law Journal* 1:15–26. 10.17159/1727-3781/1998/V1I1A2897.

[CR53] National Academies of Sciences, Engineering, and Medicine. 2015. *Improving diagnosis in health care*. Washington DC: The National Academies Press.

[CR54] Norgeot, B., B. Glicksberg, and A. Butte. 2019. A call for deep-learning healthcare. *Nature Medicine* 25: 14–18. 10.1038/s41591-018-0320-3. 10.1038/s41591-018-0320-3 30617337

[CR55] O’Neill, O. 1989. *Constructions of reason: Explorations of Kant’s practical philosophy*. Cambridge: Cambridge University Press.

[CR56] O’Neill, O. 2015. *Constructing authorities: Reason, politics and interpretation in Kant’s philosophy*. Cambridge: Cambridge University Press.

[CR57] Osheroff, J.A., J.M. Teich, B. Middleton, E.B. Steen, A. Wright, and D.E. Detmer. 2007. A roadmap for national action on clinical decision support. *Journal of the American Medical Informatics Association: JAMIA* 14 (2): 141–145. 10.1197/jamia.M2334. 17213487 10.1197/jamia.M2334 PMC2213467

[CR58] Oyebode, F. 2006. Clinical errors and medical negligence. *Advances in Psychiatric Treatment* 12 (1): 221–227. 10.1159/000346296.

[CR59] Oyowe, Anthony O. 2013. Strange bedfellows: Rethinking ubuntu and human rights in South Africa. *African Human Rights and Law Journal* 13:103–124. https://www.ahrlj.up.ac.za/images/ahrlj/2013/ahrlj_vol13_no1_2013_anthony_o_oyowe.pdf. Accessed 21 Apr 2025.

[CR60] Ploug, T., and S. Holm. 2020. The right to refuse diagnostics and treatment planning by artificial intelligence. *Medicine, Health Care, and Philosophy* 23 (1): 107–114. 10.1007/s11019-019-09912-8. 31359302 10.1007/s11019-019-09912-8

[CR61] Ross, C., and I. Swelitz. 2017. IBM pitched its Watson supercomputer as a revolution in cancer care. It’s nowhere close. *StatNews*, 5 September 2017. https://www.statnews.com/2017/09/05/watson-ibm-cancer. Accessed 20 Sept 2023.

[CR62] Salloch, S., and A. Eriksen. 2024. What are humans doing in the loop? Co-reasoning and practical judgment when using machine learning-driven decision aids. *The American Journal of Bioethics* 24 (9): 67–78. 10.1080/15265161.2024.2353800. 10.1080/15265161.2024.2353800 38767971

[CR63] Sharkey, N., and A. Sharkey. 2013. Robotic surgery: On the cutting edge of ethics. *Computer* 46 (1): 56–64. 10.1109/MC.2012.424.

[CR64] Smith, A. 2003. Do you believe in ethics? Latour and Ihde in the trenches of the Science Wars (Or: Watch out, Latour, Ihde’s got a gun). In *Chasing technoscience: Matrix for materiality*, ed. D. Ihde and E. Selinger, 182–194. Bloomington: Indiana University Press.

[CR65] Stewart, M., Brown, J., Weston, W., et al. 2006. *Patient-centred medicine: transforming the clinical method.* Oxford: Radcliffe Medical Press: Oxford

[CR66] Sun, Y.S., Z. Zhao, Z.N. Yang, F. Xu, H.J. Lu, Z.Y. Zhu, and H.P. Zhu. 2017. Risk factors and preventions of breast cancer. *International Journal of Biological Sciences* 13 (11): 1387–1397. 10.7150/ijbs.21635. 29209143 10.7150/ijbs.21635 PMC5715522

[CR67] Suthaharan, S. 2016. *Machine learning models and algorithms for big data classification*. Cham: Springer Nature..

[CR68] Thevenot, J., M.B. López, and A. Hadid. 2018. A survey on computer vision for assistive medical diagnosis from faces. *IEEE Journal of Biomedical and Health Informatics* 22 (5): 1497–1511. 10.1109/JBHI.2017.2754861. 28991753 10.1109/JBHI.2017.2754861

[CR69] Thompson, K., B. Venkatesh, and S. Finfer. 2019. Sepsis and septic shock: Current approaches to management. *Internal Medicine Journal* 49 (2): 160–170. 10.1111/imj.14199. 30754087 10.1111/imj.14199

[CR70] Topol, E. 2019. High-performance medicine: The convergence of human and artificial intelligence. *Nature Medicine* 25: 44–56. 10.1038/s41591-018-0300-7. 10.1038/s41591-018-0300-7 30617339

[CR71] Tutu, D. 1999. *No future without forgiveness*. New York: Random House.

[CR72] Ugar, E.T., and N. Malele. 2024. Designing AI for mental health diagnosis: Challenges from sub-Saharan African value-laden judgements on mental health disorders. *Journal of Medical Ethics* 50 (9): 592–595. 10.1136/jme-2023-109711. 38373829 10.1136/jme-2023-109711

[CR73] van de Poel, Ibo. 2020. Embedding values in artificial intelligence (AI) systems. *Minds and Machines* 30:385–409. 10.1007/s11023-020-09537-4.

[CR74] Verbeek, P.P. 2005. *What things do: Philosophical reflections on technology, agency, and design*. University Park: Penn State University Press.

[CR75] Verbeek, P.P. 2008. Obstetric ultrasound and the technological mediation of morality: A postphenomenological analysis. *Human Studies* 31: 11–26. 10.1007/s10746-007-9079-0.

[CR76] Waaijer, L., M.D. Filipe, J. Simons, C.C. van der Pol, T. de Boorder, P.J. van Diest, and A.J. Witkamp. 2020. Detection of breast cancer precursor lesions by autofluorescence ductoscopy. *Breast Cancer* 28:119–129. 10.1007/s12282-020-01136-6. 10.1007/s12282-020-01136-6 PMC7796885 32725533

[CR77] Warren, M. 1973. On the moral and legal status of abortion. *Natural Law Forum* 57:43–61. https://rintintin.colorado.edu/~vancecd/phil215/Warren.pdf. Accessed 21 Apr 2025. 10.5840/monist197357133 11661013

[CR78] Whitney, N. 2009. Near-drowning, futility, and the limits of shared decision-making. In *Ethical dilemmas in paediatrics: Cases and commentaries*, ed. R. Frankel, A. Goldworth, A. Rorty, and A. Silverman, 95–107. New York: Cambridge University Press.

[CR79] Wiredu, K. 1996. *Cultural universals and particulars: An African perspective*. Bloomington: Indiana University Press.

[CR80] Zhou, Yishan, and David Danks. 2020. Different “intelligibility” for different folks. In *Proceedings of the AAAI/ACM Conference on AI, Ethics, and Society*, 194–199. 10.1145/3375627.3375810.

